# The NKD1/Rac1 feedback loop regulates the invasion and migration ability of hepatocarcinoma cells

**DOI:** 10.1038/srep26971

**Published:** 2016-05-27

**Authors:** Jie Li, Sheng Zhang, Qing Hu, Kang Zhang, Jianbin Jin, Xuqing Zheng, Zhenyu Yin, Xiaomin Wang

**Affiliations:** 1 Department of Hepatobiliary Surgery, ZhongShan Hospital Xiamen University, Xiamen, China; 2 Fujian Provincial Key Laboratory of Chronic Liver Disease and Hepatocellular Carcinoma (Xiamen University Affiliated ZhongShan Hospital), Xiamen, China

## Abstract

Hepatocellular carcinoma (HCC) is complicated by aggressive migration and invasion, which contribute to the increased mortality of HCC patients. The NKD1 protein is abnormally expressed in many neoplasms and plays an important role in tumor progression. However, the regulation and underlying molecular mechanisms of NKD1 in HCC cell invasion and migration remain poorly understood. In the present study, ectopic expression of NKD1 in HCC cells attenuated migration and invasion *in vitro* and *in vivo* by down-regulating Rac1 expression level and activity, which affected the HCC cell cytoskeleton and E-cadherin expression. Mechanistic studies showed that NKD1 interacted with Rac1 in the cytoplasm and promoted its degradation by the ubiquitin-proteasome pathway. Over-expression of Rac1 enhanced the transcription of the *NKD1* gene and protein expression conversely owing to its negative regulation of EZH2. Analysis of clinical samples showed that abnormal expression of NKD1 and Rac1 was associated with the poor prognosis of HCC patients. In summary, our data indicate a new role for NKD1 as a regulator of HCC cell invasion and migration via a feedback loop involving Rac1.

Hepatocellular carcinoma (HCC) is one of the most common malignancies and the third leading cause of tumor related death worldwide after gastric and esophageal cancers^1^,^2^. It is characterized by recurrence, metastasis, and poor prognosis^3^. Although surgery and liver transplantation have been successfully used to control some cases of early HCC, recurrence and metastasis still occur in 30–40% of patients after surgery^4^,^5^. Furthermore, metastasis is the main cause of mortality in patients with HCC^6^. Hence, a better understanding of the metastatic process could help identify new therapeutic strategies to control the disease.

Accumulating evidence indicates that NKD1 antagonizes Wnt signaling by preventing the nuclear accumulation of β-catenin^7^,^8^. However, activation of the Wnt/β-catenin signaling pathway results in the up-regulation of downstream genes such as NKD1^9^. NKD1 functions in a negative feedback loop, as it is induced in response to Wnt signaling and acts to oppose the signaling pathway.

Dysregulation of NKD1 has been reported in many types of neoplasms. The NKD1 mRNA level is increased in colorectal adenomas^10^ and hepatoblastoma^11^ whereas it is decreased in HCC primary tumor tissues^12^. In addition, down-regulation of NKD1 is correlated with histological grade and estrogen receptor expression in breast cancer^13^. Loss of NKD1 protein expression is correlated with lymph node metastasis in lung adenocarcinoma^14^ and a poor prognosis in non small cell lung cancer (NSCLC)^15^. Stancikova *et al.* showed that NKD1 can serve as a reliable marker of intestinal and liver tumors that display aberrant Wnt/β-catenin signaling^16^. However, the function and mechanism of NKD1 in HCC cell invasion and migration has not been documented in detail. Furthermore, methylation of NKD1, associated with an increased risk of epithelial ovarian cancer progression and a higher risk of death^17^,^18^, is observed in 11.7% (23/196) of human gastric cancer patients^19^,^20^. Enhancer of zeste homolog 2 (EZH2) occupancy on the NKD1 promoter is associated with reduced expression of NKD1^12^. Rac1, which has been widely implicated in cytoskeleton rearrangement, cell adhesion and metastasis^21^,^22^, positively regulates NKD1 levels in colorectal cancer^23^. Taken together, these findings indicate that dysregulation of NKD1 in tumors is possibly driven by as yet un-described mechanisms in addition to the Wnt signaling pathway and epigenetics.

Our previous study showed that NKD1 protein is down regulated in HCC tissues and correlated with poor differentiation, tumor size, and intra- or extra-hepatic metastasis^24^. To improve our knowledge of the function and mechanism of NKD1 in HCC, we used gain-of-function experiments and showed that the up-regulation of NKD1 inhibited HCC cell migration and invasion *in vivo* and *in vitro* via Rac1. In addition, we showed that NKD1 co-localized and interacted with Rac1 in the cytoplasm and promoted its degradation through the ubiquitin-proteasome pathway. We showed that Rac1 positively regulated NKD1 expression via EZH2. Finally, we found that abnormal expression of NKD1 and Rac1 in clinical samples was associated with poor prognosis in HCC patients. Our results provide evidence that NKD1 is a negative regulator of HCC cell invasion and migration via a feedback loop involving Rac1.

## Results

### NKD1 expression was negatively associated with HCC cell invasion and metastasis *in vitro*

**Figure 1:**
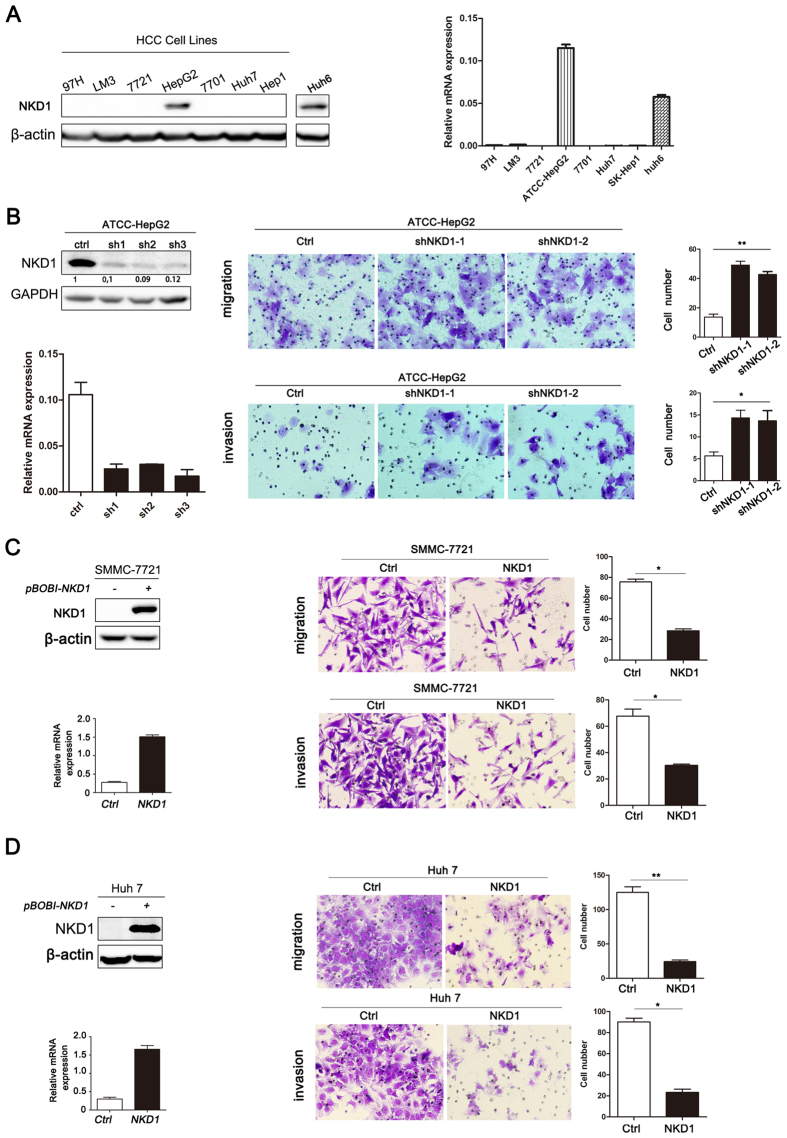
NKD1 negatively associated with HCC cell invasion and metastasis. (**A**) NKD1 protein and mRNA were almost undetectable in many cells except a moderate expression in HepG2 cell, and a separate detection was did in Huh6 cell as a positive control. (**B**) Down-regulation of NKD1 promoted HCC cell migration and invasion. (**C**,**D**) Over-expression of NKD1 repressed HCC cell invasion and migration. (*P < 0.05; **P < 0.01).

We previously reported that NKD1 protein is down-regulated in HCC tissues^24^. In the present study, we examined the NKD1 expression pattern in HCC cell lines, which showed that NKD1 was almost undetectable at the protein and mRNA levels in many cells, except for moderate expression in HepG2 cells (Fig. 1A). The Huh6 cell line showed a relatively higher level of NKD1 protein and mRNA, which was consistent with previous observations^11^. Abnormal expression of NKD1 is correlated with HCC tumor intra- or extra-hepatic metastasis, which implies that NKD1 might be associated with HCC cell invasion and metastatic ability. To further validate our assumption, we depleted endogenous NKD1 via a lentivirus vector based shRNA approach in HepG2 cells. Migration and invasion experiments showed that down-regulation of NKD1 significantly promoted HCC cell migration and invasion (Fig. 1B). Conversely, when NKD1 plasmid was cloned into a lentiviral vector and stably transfect into the SMMC-7721 and Huh 7 cell lines, invasion and migration data showed the opposite results (Fig. 1C,D). In summary, our data indicated that NKD1 expression was negatively associated with HCC cell invasion and metastasis *in vitro*.

### NKD1-mediated regulation of HCC cell invasion and migration is dependent on Rac1

**Figure 2:**
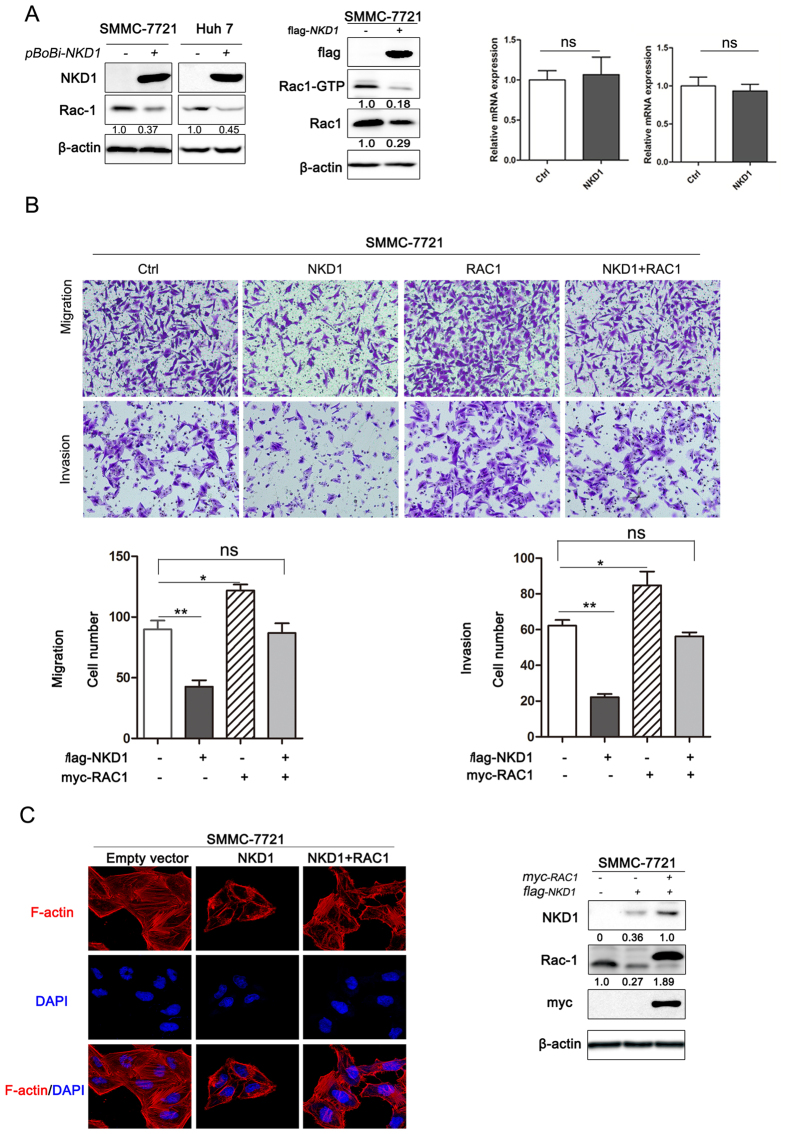
NKD1 inhibits HCC cell invasion and migration is dependent on Rac1. (**A**) Ectopic expression of NKD1 in SMMC-7721 and Huh7 cells decrease the Rac1 protein activity along with decrease of the quantification of Rac1, whereas it had no effect on Rac1 mRNA levels. (**B**) NKD1 over-expression-mediated inhibition of the invasion and migration of HCC cells was strongly restored by the expression of Rac1. (**C**) The cytoskeletal rearrangement of SMMC-7721 with NKD1 over-expression group was less than that with the control group. However, this weakness was strongly restored with the over-expression of Rac1. (*P < 0.05; **P < 0.01).

Although the effect of NKD1 on the negative regulation of HCC cell invasion and metastasis is clear, the underlying molecular mechanism remains unknown. Rac1, as an important small GTPase, has a pivotal role in cancer cell migration, invasion, and metastasis^21^,^22^. In addition, Rac1 has been shown to induce NKD1 mRNA expression in colorectal cancer^23^. Based on our present results and previous reports, we hypothesized that Rac1 may be modulated by NKD1. Ectopic expression of NKD1 in SMMC-7721 and Huh7 cells down-regulated Rac1 protein expression and its protein activity as well, although no changes were detected at the mRNA level (Fig. 2A). Next, we sought to determine whether the effect of NKD1 on HCC cell invasion and migration could be attributed to Rac1 induction. To this end, a rescue experiment was designed and performed. As shown in Fig. 2B, NKD1 over-expression induced inhibition of invasion and migration was strongly restored by the expression of Rac1. In addition, cell cytoskeleton rearrangements are regulated by Rac1 and provide a critical process for the invasive and metastatic spread of tumor cells. To assess whether NKD1 plays a role in the rearrangements of the HCC cell cytoskeleton, we detected F-actin levels by immunofluorescence. As shown in Fig. 2C, the cytoskeletal rearrangement of SMMC-7721 with NKD1 over-expression group was less than that with the control group. However, this weakness was strongly restored with the over-expression of Rac1. Taken together, these results suggested that NKD1 over-expression in HCC cells down-regulated Rac1, resulting in cell cytoskeleton rearrangement and the inhibition of HCC cell invasion and migration.

### NKD1 interacts with Rac1 and promotes its degradation through the ubiquitin-proteasome pathway

**Figure 3:**
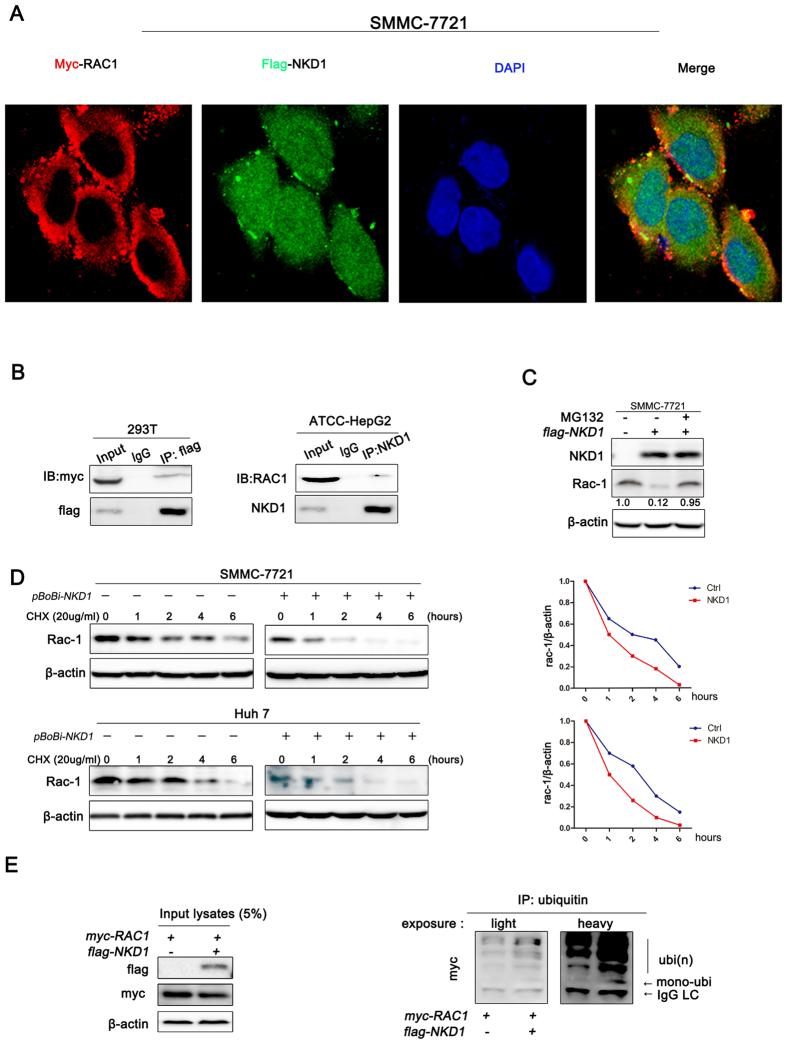
NKD1 interacts with Rac1 and promotes its degradation via ubiquitin-proteasome pathway. (**A**) NKD1 slightly co-localized with Rac1 in the cytoplasm which is displayed as yellow areas. (**B**) Co-immunoprecipitation experiments indicated that NKD1 interacts with Rac1 in 293T and HepG2 cells. (**C**) MG132 treatment prevented NKD1-mediated Rac1 degradation. (**D**) NKD1 significantly shortened the half-life of Rac1. (**E**) The addition of NKD1 to Rac1-transfected HCC cells increased Rac1 poly-ubiquitination.

To investigate the possible interaction of NKD1 with Rac1, we assessed the intracellular localization of NKD1 and Rac1 using immunofluorescence laser scanning confocal microscopy. The results showed that a small number of NKD1 co-localized with Rac1 in the cytoplasm of SMMC-7721 cells (Fig. 3A). In addition, co-immunoprecipitation results indicated that NKD1 slightly interacted with Rac1 in 293T and HepG2 cells (Fig. 3B). Our results showed that NKD1 did not significantly reduce Rac1 transcription, whereas it altered its protein level. Polyubiquitination is a major mechanism for protein degradation by the proteasome. Therefore, we treated NKD1 over-expressing cells with the proteasome inhibitor MG132 and observed whether the NKD1-mediated Rac1 down-regulation could be rescued with the addition of MG132. The results showed that MG132 treatment prevented NKD1-mediated Rac1 degradation (Fig. 3C). Next, to evaluate the effect of NKD1 on Rac1 protein stability, SMMC-7721 and Huh7 cells were treated with cycloheximide (CHX), an inhibitor of protein synthesis. Western blot analysis indicated that NKD1 significantly shortened the half-life of the Rac1 protein (Fig. 3D). Furthermore, the addition of NKD1 to Rac1-transfected cells increased Rac1 polyubiquitination (Figs 3E and S1). These data suggested that NKD1 interacts with Rac1 and promotes its degradation by inducing polyubiquitination-mediated proteasomal degradation.

### Rac1 activates NKD1 transcription in HCC cells by down-regulating EZH2 expression

**Figure 4:**
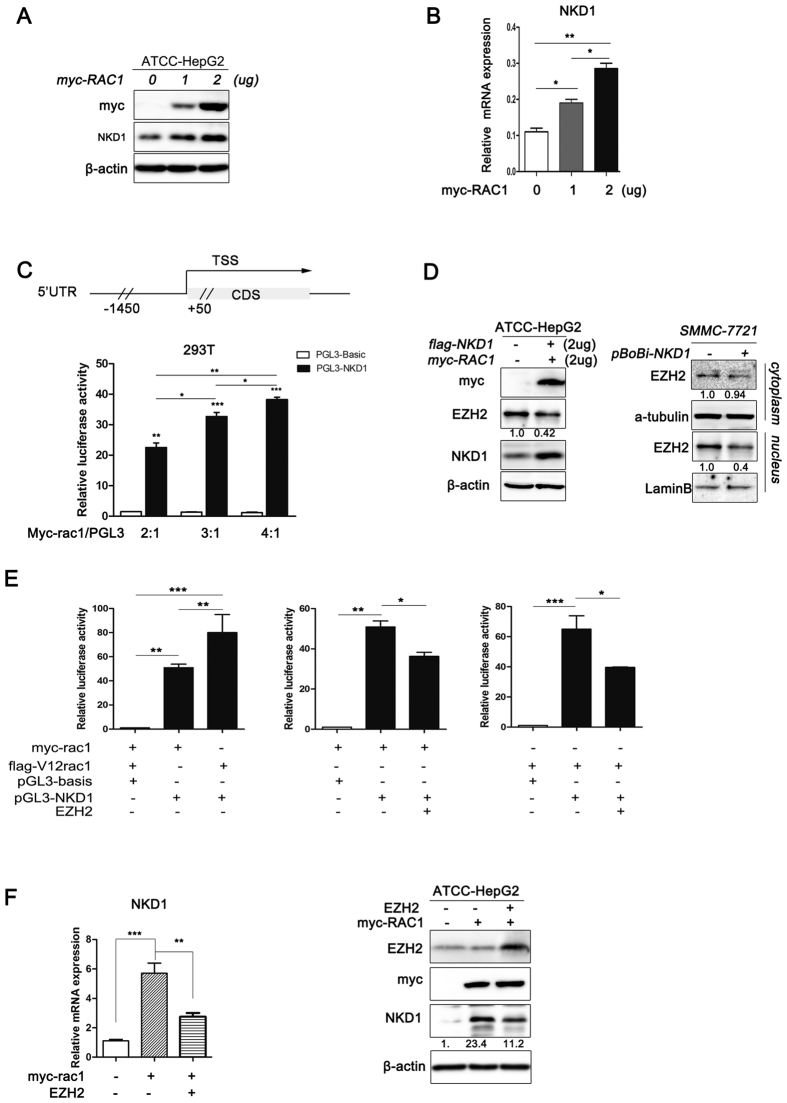
Rac1 activates NKD1 transcription by down-regulating EZH2 expression. (**A**,**B**) Rac1 up-regulated NKD1 at the mRNA and protein levels in HCC cells. (**C**) Rac1 significantly induced the activity of the NKD1 reporter gene. (**D**) Rac1 over-expression decreased the protein level of EZH2 (from 1.0 to 0.42), especially its affection on nucleus EZH2 protein express level (from 1.0 to 0.4). (**E**) Over-expression Rac1 or V12Rac1 could enhance NKD1 promoter activity, but the effects could be weakened partially when over-expression EZH2 in over-expression Rac1 or V12Rac1 cells simultaneously. (**F**) Rac1 increase NKD1 mRNA and protein expression partially through EZH2. (*P < 0.05; **P < 0.01).

A previous study showed that Rac1 positively regulates NKD1 mRNA levels in colorectal cancer^23^. To determine whether Rac1 has a similar effect on HCC cell lines, we measured NKD1 mRNA and protein levels after ectopic expression of Rac1 by real-time PCR and western blotting, respectively. The results showed that Rac1 up-regulated NKD1 at the mRNA and protein levels in HCC cells (Fig. 4A,B). Next, we examined the effect of Rac1 on NKD1 transcriptional activity using an NKD1-luciferase reporter system. As shown in Fig. 4C, Rac1 significantly induced the activity of the NKD1 reporter gene. EZH2 has been shown to inhibit NKD1 transcriptional activity in HCC cells^12^. Therefore, we speculated that Rac1 promotes NKD1 transcription because of its effect on EZH2. To test this hypothesis, the expression level of EZH2 was detected by western blotting. Rac1 over-expression decreased the protein level of EZH2, especially its affection on nucleus EZH2 protein express level. (Fig. 4D). Over-expression Rac1 or V12Rac1 (a constitutively active form of Rac1) could enhance NKD1 promoter activity, but the effects could be weakened partially when over-expression EZH2 in over-expression Rac1 or V12Rac1 cells simultaneously (Fig. 4E). What’s more, Rac1 increase NKD1 mRNA and protein expression partially through EZH2 (Fig. 4F). These results indicated that the Rac1-mediated down-regulation of EZH2 expression might contribute to the up-regulation of NKD1 transcriptional activity.

### Ectopic expression of NKD1 inhibited HCC metastasis *in vivo*

**Figure 5:**
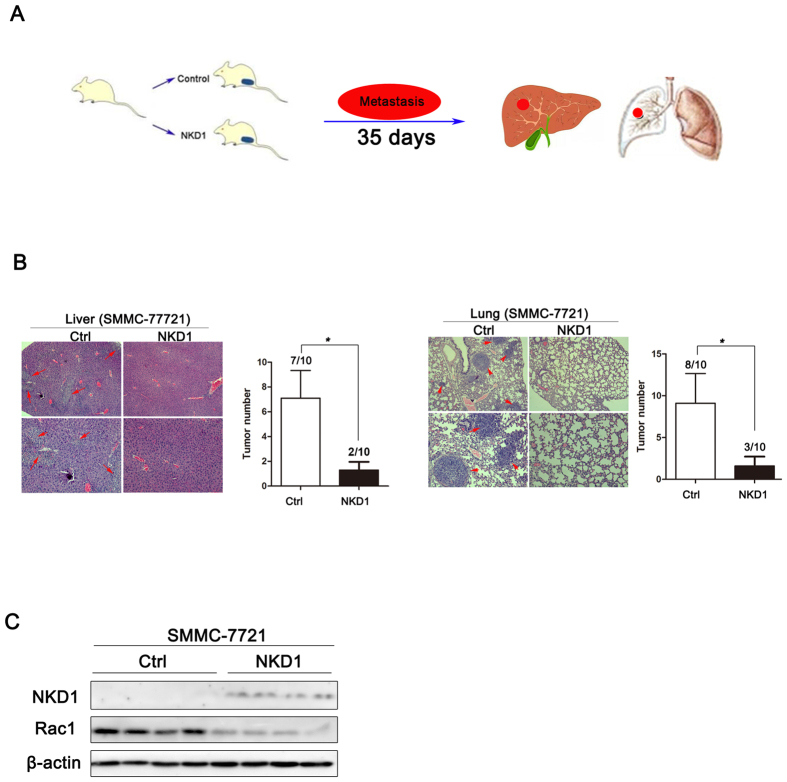
Ectopic expression of NKD1 inhibited HCC metastasis *in vivo*. (**A**) A picture was drawn by us which describe the procedure of generating the nude mouse xenograft model. (**B**) The mice injected with SMMC-7721+Ctrl cells displayed more pulmonary and intra-hepatic micro-metastatic nodules and a higher metastasis rate than mice injected with SMMC-7721+NKD1. (**C**) Western blots analysis of the expression of NKD1 and Rac1 protein in SMMC-7721+Ctrl (the left four lanes) and SMMC-7721+NKD1 tumors (the right four lanes). (*P < 0.05).

Next, we examined the effect of NKD1 on HCC cell migration and invasion in mice using a heterotopic xenograft model, which was generated via the subcutaneous injection of SMMC-7721+Ctrl and SMMC-7721+NKD1 cells into nude mice. At 35 days after implantation, treated mice were sacrificed by euthanasia and the metastatic nodules in lung and liver were counted (Fig. 5A). As shown in Fig. 5B, the mice injected with SMMC-7721+Ctrl cells displayed more pulmonary and intra-hepatic micro-metastatic nodules and a higher metastasis ratio than mice injected with SMMC-7721+NKD1. To correlate the biological response with the mechanisms identified in the HCC cell system, Rac1 protein levels were detected by western blotting. Consistently, ectopic expression of NKD1 significant decreased Rac1 protein level in transplanted tumor tissue (Fig. 5C). Collectively, these data suggested that ectopic expression of NKD1 inhibits HCC cell metastasis *in vivo.*

### Abnormal expression of NKD1 and Rac1 is associated with poor prognosis in HCC patients

**Figure 6:**
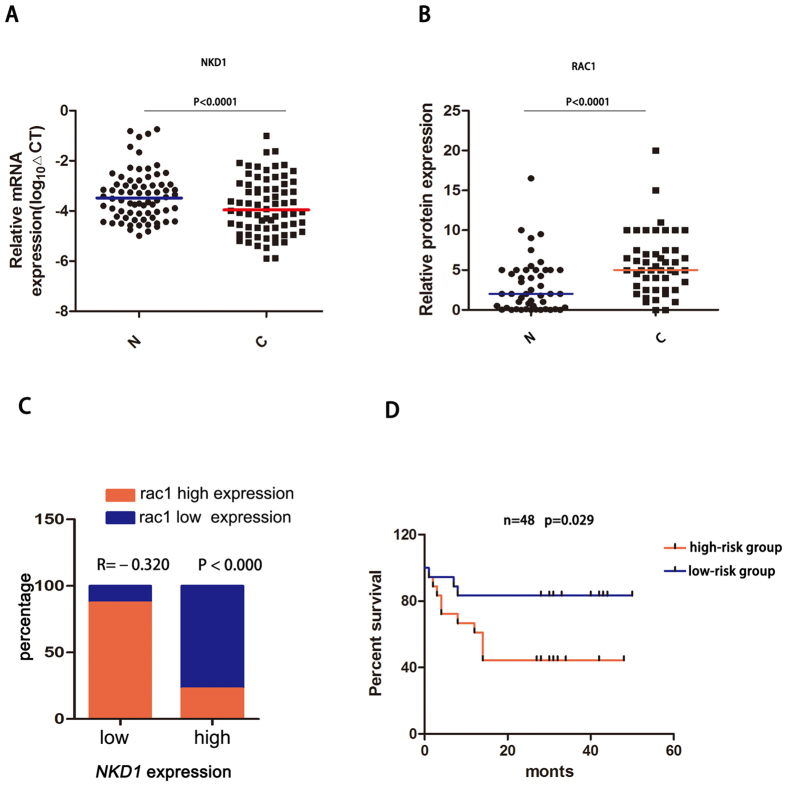
Abnormal expression of NKD1 and Rac1 associated with a poor prognosis. (**A**,**B**) NKD1 mRNA was down-regulated in HCC tissues compared with non-tumor tissues, whereas Rac1 protein was up-regulated in HCC tissues (P < 0.0001). (**C**) There was a negative correlation between NKD1 expression levels and Rac1 in HCC clinical samples (P = 0.00023, R = −0.32). (**D**) Survival analysis showed that the patients with a relatively lower NKD1 and higher Rac1 expression (regarded as the high risk group) had a shorter overall survival time (P = 0.029).

Finally, we explored the expression patterns and clinical significance of NKD1 and Rac1 in HCC patient tissues. Our previous study showed that NKD1 protein is down-regulated in HCC tissues compared with adjacent non-cancerous tissues^24^. In the present study, the mRNA expression of NKD1 and the protein level of Rac1 in paired HCC tissues were analyzed by real-time PCR and western blotting, respectively. As shown in Fig. 6A,B, NKD1 was down-regulated at the mRNA level in HCC tissues compared with non-tumor tissues, whereas Rac1 was up-regulated in HCC tissues, although no difference was found in the Rac1 mRNA level between HCC and non-tumor tissues (Fig. S2). In addition, there was a negative correlation between NKD1 expression levels and Rac1 in HCC clinical samples (P = 0.00023, R = −0.32, Fig. 6C). Moreover, the patients with relatively lower NKD1 and higher Rac1 expression in tumor tissues compared with paired non-tumor (regarded as the high risk group) had a shorter overall survival time (Fig. 6D, P = 0.029), indicating that abnormal expression of NKD1 and Rac1 can predict a poor clinical outcome in HCC patients.

## Discussion

Several aberrant intracellular signaling pathways have been identified in HCC, including the canonical Wnt signaling pathway^25^. Moreover, studies have shown that invasion and metastasis are the main reasons for the high mortality and recurrence rate of HCC^26^. NKD1, which is abnormally expressed in many neoplasms, suppresses the Wnt signaling pathway by sequestering disheveled protein (Dvl) and preventing the nuclear accumulation of β-catenin^7^,^8^. Although the various functions of NKD1 have been extensively studied, the role of NKD1 in HCC cell migration and invasion remains relatively poorly understood. Here, we showed that NKD1 was significant down-regulated in HCC cells and negatively associated with HCC cell invasion and metastasis *in vivo* and *in vitro.* This effect of NKD1 is mediated by the modulation of Rac1. Furthermore, mechanistic studies uncovered a novel function of NKD1 based on its interaction with Rac1 in the cytoplasm, which promoted Rac1 degradation through the ubiquitin-proteasome pathway, leading to the rearrangement of the cell cytoskeleton. Our results indicate that Rac1 could reversely promote NKD1 transcription in HCC cells by down-regulating EZH2 expression, establishing a feedback loop between NKD1 and Rac1. Clinical sample analysis confirmed that abnormal expression of NKD1 and Rac1 was associated with poor prognosis in HCC patients.

In the present study, we confirmed that NKD1 mRNA and protein were down-regulated in HCC tissues compared with non-tumor tissues, which was consistent with previous findings by Cheng *et al.*^12^, but inconsistent with reports on colorectal adenomas^10^ and NSCLC^15^. Although it is a general concept that certain indicators are over-expressed or down-regulated in tumors consistently, there are exceptions, such as NKD1. This contradictory expression pattern may be related to tumor and tissue specificity, and caused by complex signaling pathways as well. In addition, increasing evidence indicates that NKD1 gene transcription is enhanced when the Wnt/β-catenin signaling pathway is activated^9^. Meanwhile, aberrant activation of the Wnt signaling pathway is observed in the S1 subtype of HCC^27^. Hence, NKD1 mRNA is believed to be up-regulated in HCC. The contradictory findings could be attributed to the fact that NKD1 transcription is regulated not only by the Wnt signaling pathway, but also by other possibly unknown molecular mechanisms, which needs further exploration. At least, it is unquestionable that NKD1 gene transcription is regulated by epigenetics^28^.

NKD, a homolog of NKD1 in Drosophila, contains a nuclear-localization sequence and binds to the nuclear import adaptor Importin-α3 to antagonize Wnt/β-catenin signaling^28^. In the present study, ectopic expression of NKD1 in SMMC-7721 cells resulted in the detection of NKD1 in the nucleus by immunofluorescence. Considering the aforementioned results, we surmise that NKD1 may be involved in nuclear import and play a role as a transcriptional regulator. We are currently testing the possible mechanism via gene expression profiling.

Evidence indicates that Rac1 positively regulates NKD1 mRNA expression in colorectal cancer^23^. However, the underlying mechanism remains unclear. In the present study, we initially found that EZH2, which binds to the NKD1 promoter and is associated with the down-regulation of NKD1^12^, was down-regulated by ectopic expression of Rac1 in HCC cells. Our results provide clues to improve our understanding of Rac1 modulation of NKD1 expression, not only through the Wnt/β-catenin pathway^23^, but also by decreasing EZH2 protein levels. However, the mechanism underlying the Rac1 regulation of EZH2 protein expression, through direct or indirect means, remains to be elucidated.

E-cadherin, a dynamic biomarker of the EMT process, is crucial for the development of tumor metastasis^29^ and is associated with HCC carcinogenesis^30^. Recent publications reported that Rac1 could inhibit E-cadherin mediated adherens junctions in pancreatic carcinoma cells^31^. We therefore investigated whether NKD1 has a similar effect on E-cadherin expression in our experimental model. Our results showed that adenoviral-mediated NKD1 over-expression was associated with increased E-cadherin protein expression (Fig. S3). These data suggest that NKD1 over-expression in HCC cells induces a decrease of Rac1 protein levels and, as a consequence, the up-regulation of E-cadherin expression, which results in lower cell invasion and migration ability.

**Figure 7:**
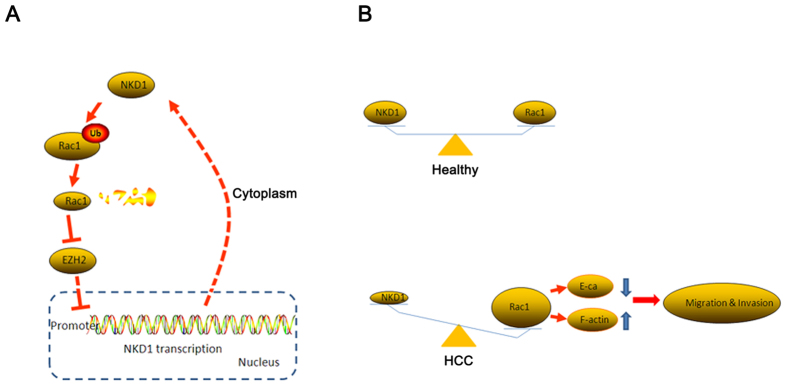
A proposed model depicting the mechanism by which NKD1/Rac1 feedback loop regulates the invasion and migration ability of hepatocarcinoma cells. (**A**) A possible model of the feedback loop between Rac1 and NKD1. (**B**) A balance exists between NKD1 and Rac1 in healthy people, whereas it is abolished or inhibited by other pathways or mechanisms in HCC patients.

Based on our data, we drafted a possible model to explain the feedback loop between Rac1 and NKD1. NKD1 combines with Rac1 in the cytoplasm and promotes its degradation through the ubiquitin-proteasome pathway. The down-regulation of Rac1 impairs its inhibition of EZH2 protein expression. Consequently, NKD1 transcription is inactive owing to the occupancy of EZH2 on its promoter (Fig. 7A). Furthermore, we speculated on the existence of a balance between NKD1 and Rac1 in healthy people. In HCC patients, the balance is either abolished or inhibited by other pathways or mechanisms. NKD1 down-regulation in HCC impairs its inhibition of Wnt signaling, resulting in the constitutive activation of the pathway and the promotion of oncogene transcription. On the other hand, Rac1 over-expression can down-regulate E-cadherin expression and induce cell cytoskeleton rearrangements, thereby promoting HCC cell migration and invasion (Fig. 7B). However, future studies are needed to verify these hypotheses.

In conclusion, aberrant expression of NKD1 plays an important role in the process of HCC metastasis and migration via a feedback loop with Rac1. Our data open a novel avenue to investigate the molecular mechanism of HCC progression and to develop potential therapeutic strategies against HCC. Targeting NKD1 maybe be a potential useful therapeutic strategy for the prevention of HCC recurrence and metastasis.

## Methods

### Cell culture

Human liver cancer cell lines were purchased from the cell bank of Shanghai Institute of Cell Biology and cultured in medium (HyClone) supplemented with 10% fetal calf serum (Gibco), 100 IU/ml penicillin and 100 μg/ml streptomycin (Millipore).

### Western blot

Protein samples were fractionated by sodium dodecyl sulfate polyacrylamide gel electrophoresis and transferred to polyvinylidene fluoride membranes (Millipore). After blocking with 5% non-fat milk for 2 h at room temperature, the membranes were incubated with primary antibodies against NKD1 (1:1000, #2262, CST), Rac1 (1:500, 66122-1-Ig, Proteintech), EZH2 (1:1000, #5246, CST), GAPDH (1:5000, AT0010,CMCTAG), β-Actin (1:1000, AT0001, CMCTAG), E-cadherin (1:1000, #14472, CST), Flag (1:1000, F1804, Sigma), and Myc (1:500, 60003-2-Ig, Proteintech) at 4 °C overnight. Subsequently, the blots were incubated with the corresponding secondary antibody labeled with horseradish peroxidase (Yeasen) at room temperature for 2 h. Protein bands were quantified by densitometry using Image-J software and compared to the reference protein. Experiments were repeated in triplicate independently.

### Real-time PCR

RNA was extracted from tissues or cell samples using the Trizol reagent (Invitrogen) according to the manufacturer’s instructions. Primers were designed and synthesized by BGI-Tech. The sequences of the primer pairs are shown in Table 1. A dissociation procedure was performed to generate a melting curve for confirmation of amplification specificity. β-actin mRNA was quantified in parallel as the endogenous control.

### Plasmid construction and lentivirus preparation

For target gene knockdown, control and shRNA sequences (shown in Table 1) against NKD1 were cloned into the pSIREN-RetroQpuro RNA interference vector downstream from the U6 promoter. For over-expression, a 1413 bp genomic sequence of the NKD1 coding region was cloned into the backbone of the PBOBI-CMV vector downstream of the CMV promoter. In addition, constitutively active mutant Rac1-V12 plasmid was cloned into retroviral vector PCMV-flag. Then 293T cells were transfected with the above mentioned plasmids and packaging vectors by using the Turbofect Transfection Reagent (Thermo). Infected cells were cultured for selection with puromycin (InvivoGen).

### Migration and invasion assay

Migration and invasion assays were performed as previous described^32^. Briefly, 2 × 10^5^ cells were seeded in the upper chamber and 800 μl medium supplemented with 15% serum was added to the lower chamber (BD BioCoat). After 48 h in culture, migrated and invaded cells were fixed and stained with a dye solution. Five random microscopic fields per well were counted with the double-blind method.

### Rac1 Activity Assay

Lysates were made according to Rac1 Activation Magnetic Beads Pulldown Assay kits (17-10394, Merck Millipore, German). Briefly, cells were lysed in magnesium lysis buffer (MLB) supplemented with 10 μg/ml leupeptin, 10 μg/ml apoprotin, 1 mM sodium vanadate, and 1 mM sodium flouride. Lysates were then incubated with PAK-1 PBD, Magnetic beads for 45 minutes at 4 °C with gentle agitation. Pellet the beads by setting the tubes on a magnetic tube stand for a few seconds. Then the beads were washed three times with MLB. Before boiled in water for 8 min the samples were prepared for electrophoresis by adding 1× loading buffer. After resolved by 10% SDS-PAGE, proteins were transferred to PVDF membrane and GTP-bound Rac1 was identified by anti-Rac1 antibody (1:500, 66122-1-Ig, Proteintech).

### Immunofluorescence analysis

SMMC-7721 cells which were seeded onto 12-mm cover slips were fixing with 4% paraformaldehyde for 20 min, and permeabilized with 0.25% Triton-X 100, blocked with 2% bovine serum albumin. Cells were stained with primary antibodies against NKD1 (1:1000, #2262, CST), Rac1 (1:200, SC-95, SantaCruz), Phalloidin (1:750) and DAPI (1:1000) overnight at 4 °C. Images were obtained by confocal immunofluorescence microscopy (Zeiss).

### Co-immunoprecipitation

The cells were harvested and lysed in cold lysis buffer. Equal amounts of cell lysates were incubated with specific primary antibodies or control IgG overnight at 4 °C followed by the addition of 40 μl of protein A/G-agarose. The immunoprecipitated complex was washed three times with 1% Tritonx-100 in ice-cold phosphate buffer solution (PBS). The immunoprecipitates were eluted from the beads by incubation at 95 °C for 5 min. The eluted proteins were separated by SDS-PAGE and western blotting was subsequently performed with the indicated antibodies.

### Ubiquitination

Input lysates were analyzed for Rac1, NKD1, and β-actin expression by immunoblotting with myc, flag and β-actin antibodies, respectively. Cell lysates were incubated with Rac1 antibodies followed by immunoprecipitation and immunoblot analyses.

### Luciferase reporter assays

For the NKD1 reporter assays, a 1500 bp genomic sequence of the NKD1 5′-UTR (−1450–+45 bp) non-coding region was cloned into the backbone promoter region of the pGL3 vector. After 48 h, the luciferase activity was measured using a luciferase assay system (Promega) according to the manufacturer’s instructions.

### Animal studies

For the *in vivo* metastasis assays, 2 × 10^5^ cells were injected subcutaneously into the armpit of 4 week-old nude mice (10 cases for the SMCC-7721 Ctrl group and SMCC-7721 NKD1 over-expression group respectively). The mice were sacrificed via euthanasia method 35 days later. Lung and liver samples were collected for metastatic foci examination. All procedures involving experimental mice were performed in accordance with relevant protocols and regulation that were approved by the Committee for Animal Research of Xiamen University and complied with the guideline for the Care and Use of Laboratory Animals (NIH publication No. 86-23, revised 1985).

### Hematoxylin-eosin staining

Tissues were fixed with 10% neutral formalin, embedded in paraffin, and 4 μm thick sections were prepared by the pathology technologist. Then, sections were deparaffinized and hydrated with a gradient of alcohol. After soaking in PBS, sections were stained with hematoxylin for 5 minutes and eosin for 10 seconds. We had two clinical pathologists who were unaware of the experimental data evaluate the HE-stained sections and count the tumor numbers. Nearly 95% of the evaluating results were consistent in our study. And if the consensus could not be reached after the re-evaluated, a third pathologist was consulted to make the final decision. In addition, five random microscopic fields per section were counted and finally got a mean value.

### Patients and specimens

All clinical samples and follow-up information were obtained from the Chronic Liver Disease Biological Sample Bank, Department of Hepatobiliary Surgery, ZhongShan Hospital Xiamen University. Specimen collection was performed after obtaining informed consent from each patient, and the utilization of the tumor materials for research purposes was carried out in accordance with the approved guidelines and approved by the Ethics Committee (No: 20111008) of ZhongShan Hospital Xiamen University. A total of 73 HCC patients were included in our study. All of the patient’s characteristics are summarized in Table S1.

### Statistical analysis

All data were expressed as the mean ± SD and analyzed using SPSS version 21.0 (IBM). Statistical analysis was performed with two-related samples using the Wilcoxon nonparametric test for comparing two different groups. Correlation analysis was used to explore the relationship between NKD1 and Rac1. Survival curves were calculated by the Kaplan-Meier test. P value less than 0.05 was considered statistically significant.

## Additional Information

**How to cite this article**: Li, J. *et al.* The NKD1/Rac1 feedback loop regulates the invasion and migration ability of hepatocarcinoma cells. *Sci. Rep.* **6**, 26971; doi: 10.1038/srep26971 (2016).

## Supplementary Material

Supplementary Information

## Figures and Tables

**Table 1:** Primers sequences.

RT-PCR	Primer name	F:5′-3′	R:5′-3′
	NKD1	CAGCGGAGATGAGAAGAAGATG	CAAAGTCATACAGGGTGAAGGT
	β-actin	ATAGCACAGCCTGGATAGCAACGTAC	CACCTTCTACAATGAGCTGCGTGTG
	Rac1	GTTATGGTAGATGGAAAACCGG	TTGCTTTTCCCTTGTGAGTC
shRNA	NKD1-1	ccggtAGGAGAAACCACTACTTAGTTCAAGAGACTAAGTAGTGGTTTCTCCTTTTTTTACGCGTg	aattcACGCGTAAAAAAAGGAGAAACCACTACTTAGTCTCTTGAACTAAGTAGTGGTTTCTCCTa
	NKD1-2	ccggtGCCATGAACATCACCACCATTTCAAGAGAATGGTGGTGATGTTCATGGTTTTTTACGCGTg	aattcACGCGTAAAAAACCATGAACATCACCACCATTCTCTTGAAATGGTGGTGATGTTCATGGCa
	NKD1-3	ccggtGCCAAGAAGCAGCTGAAGTTTTCAAGAGAAACTTCAGCTGCTTCTTGGTTTTTTACGCGTg	aattcACGCGTAAAAAACCAAGAAGCAGCTGAAGTTTCTCTTGAAAACTTCAGCTGCTTCTTGGCa
Pbobi-cmv	NKD1	CCGCTCGAGATGGATTACAAGGATGACGACGATAAGGGGAAACTTCACTCCAAGCC	CGGAATTCCTATGTCTGGTAGAAGTGGT
	HA-UB	GCTCTAGAATGTACCCATACGACGTCCCAGACTACGCTCAGATCTTCGTGAAAACCCTT	CCGCTCGAGTTAACAGCCACCCCTCAGGCGCAGGACCA
	MYC-Rac1	GCTCTAGAATGTACCCATACGACGTCCCAGACTACGCTCAGGCCATCAAGTGTGTGGTGGT	CCGCTCGAGTTACAACAGCAGGCATTTTCT
	EZH2	TGCTCTAGAATGGGCCAGACTGGGAAGAAATC	CCGCTCGAGTCAAGGGATTTCCATTTCT
PGL3	NKD1	CCGCTCGAGTACTGAATGAAGAATTGTAAC	CATGCCATGGGCTGGGGCTGCGGCGGCC
