# Supplementary material for: The NKD1/Rac1 feedback loop regulates the invasion and migration ability of hepatocarcinoma cells
Source: Sci Rep. 2016 May 27;6:26971. doi: 10.1038/srep26971 (PMC4882592; doi:10.1038/srep26971)

Supplementary Information

The NKD1/Rac1 feedback loop regulates the invasion and migration ability of hepatocarcinoma cells

Jie Li+, Sheng Zhang+, Qing Hu, Kang Zhang, Jianbin Jin, Xuqing Zheng, Zhenyu Yin* and Xiaomin Wang *

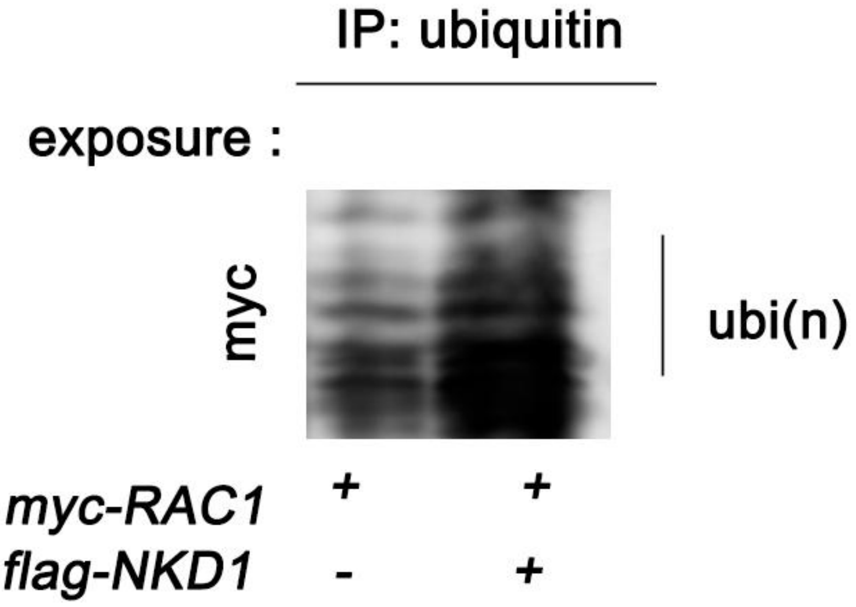

Figure S1. The addition of NKD1 to Rac1-transfected HCC cells increased Rac1 poly-ubiquitination.

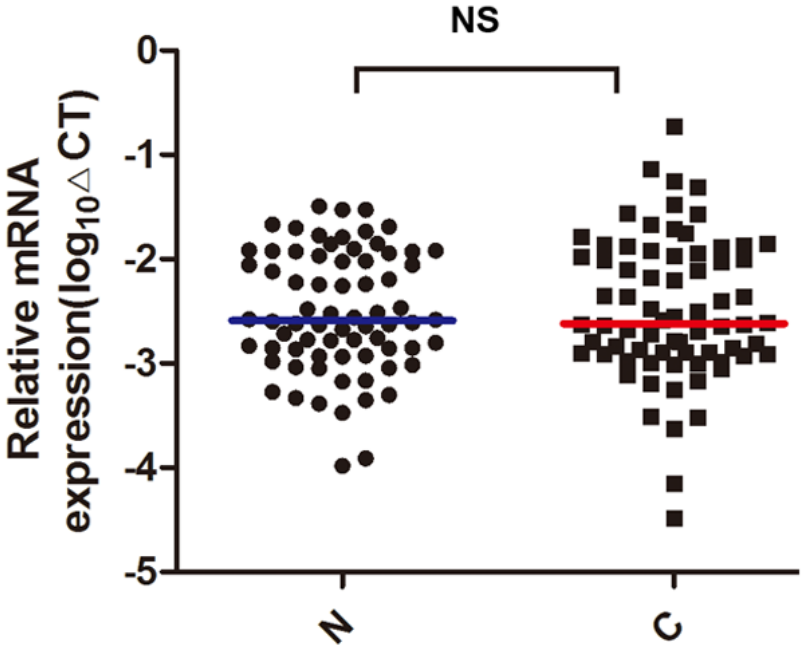

Figure S2. The expression pattern of Rac1 mRNA in paired tissues. No difference was found in Rac1 mRNA levels between HCC (C) and non-tumor tissues (N).

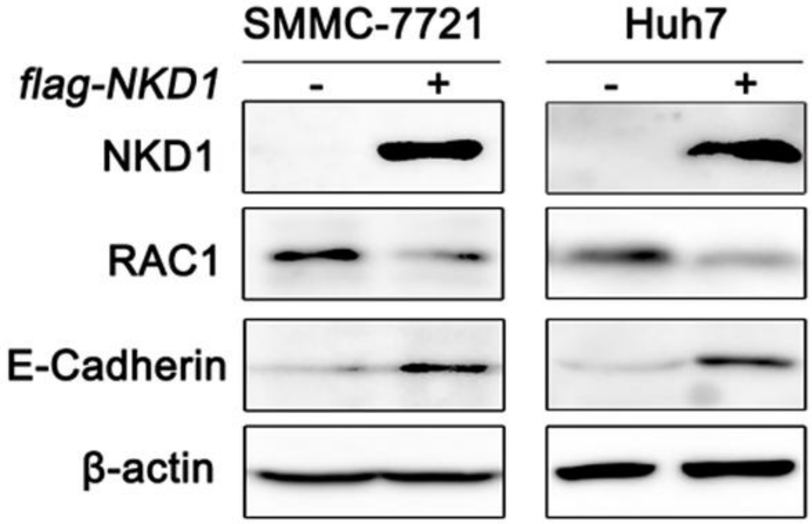

Figure S3. NKD1 was positively related with E-cadherin protein expression. NKD1 ectopic expression can increase E-cadherin protein levels in SMCC-7721 and Huh7 cells.

Table S1. HCC patients' characteristics

Characteristics	Number	%
Total	73	100
Mean age(y)	48.5	
Gender		
Male	60	82.2
Female	13	17.8
Differentiation		
Well-moderate	65	89.0
Poor	8	11.0
Portal vein tumor thrombosis		
No	30	41.1
Yes	43	58.9
Tumor size		
≥5cm	52	71.2
<5cm	21	28.8
Serum AFP level		
<400	22	30.1
≥400	51	69.9
Serum HBV level		
<1000	28	38.4
≥1000	45	61.6
Cirrhosis		
Yes	59	80.8
No	14	19.2
Fatty degeneration		
Yes	46	63.0
No	27	37.0

The full-length blots in Fig.1

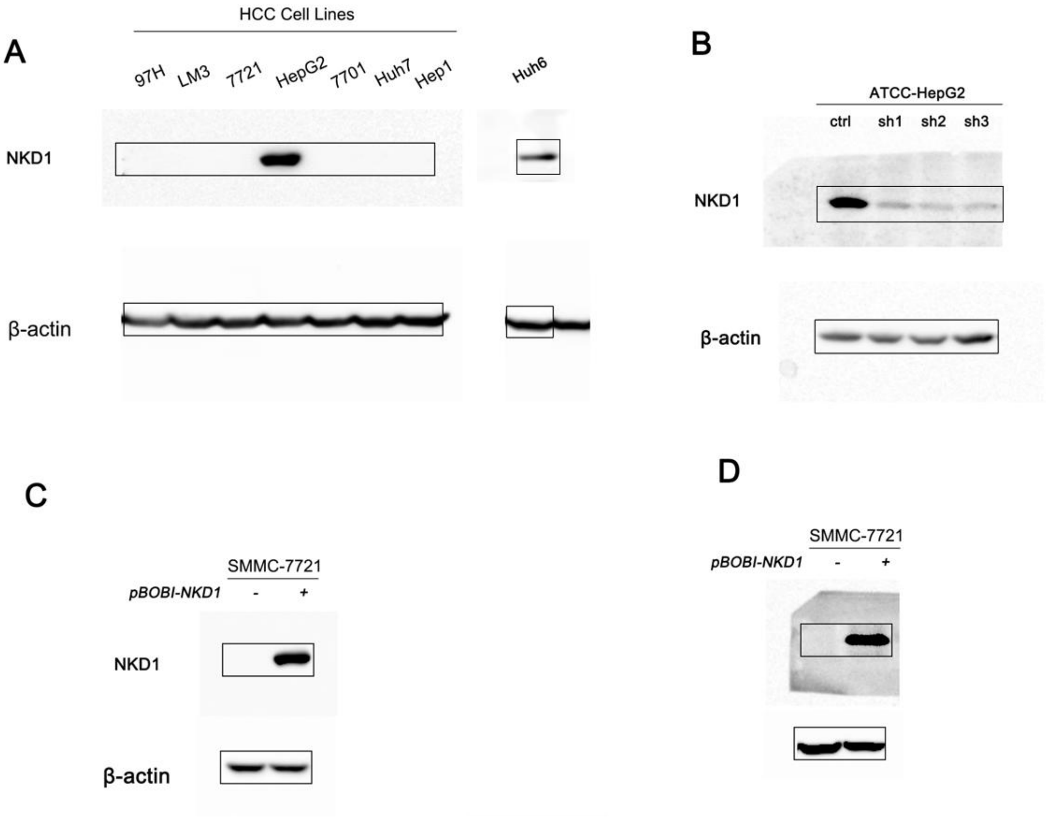

The full-length blots in Fig.2

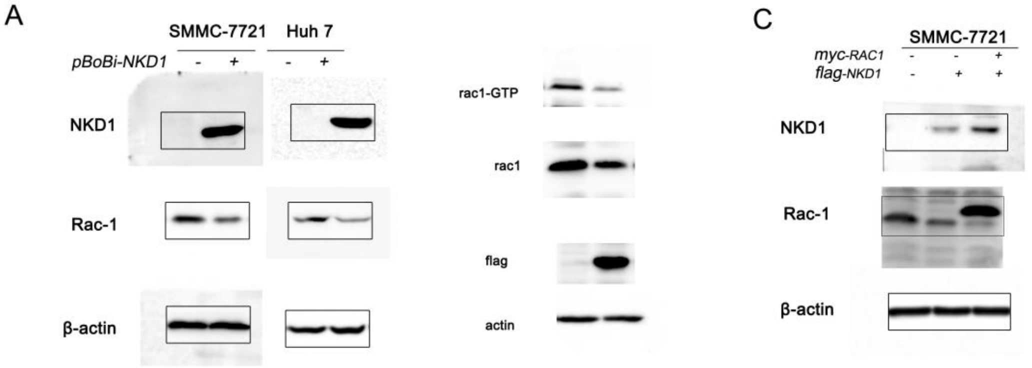

The full-length blots in Fig.3

B

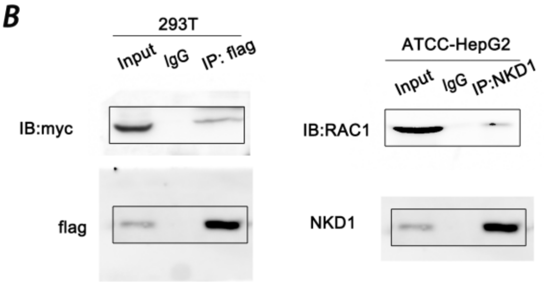

C

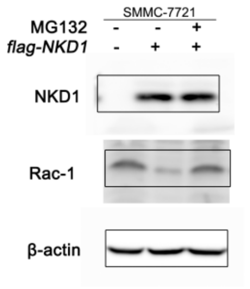

D

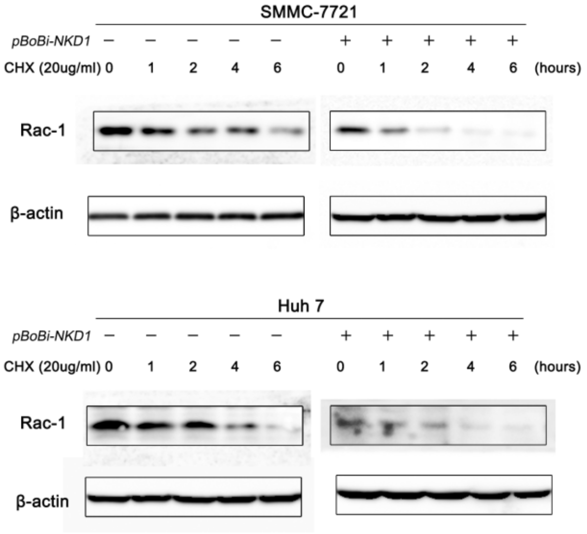

E

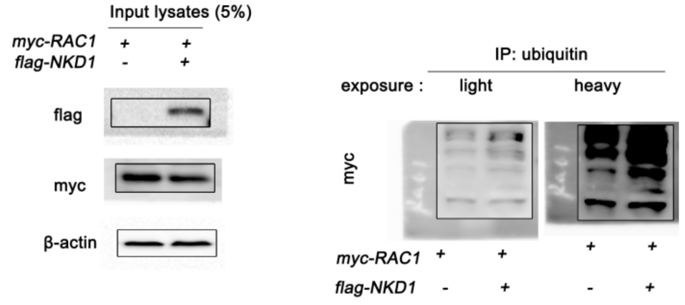

The full-length blots in Fig.4

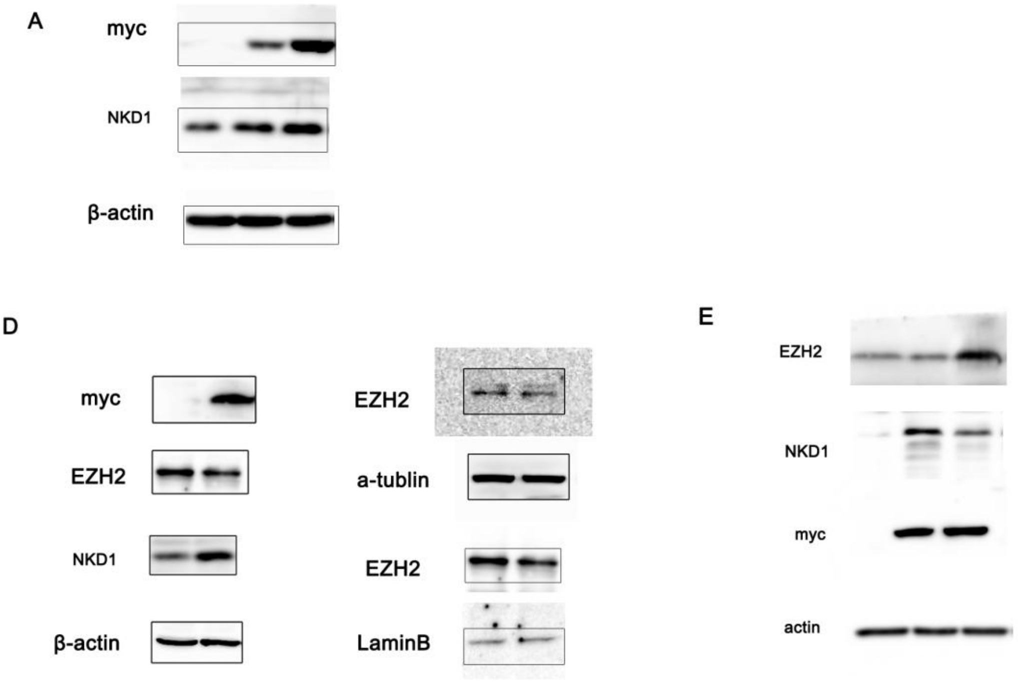

The full-length blots in Fig.5c

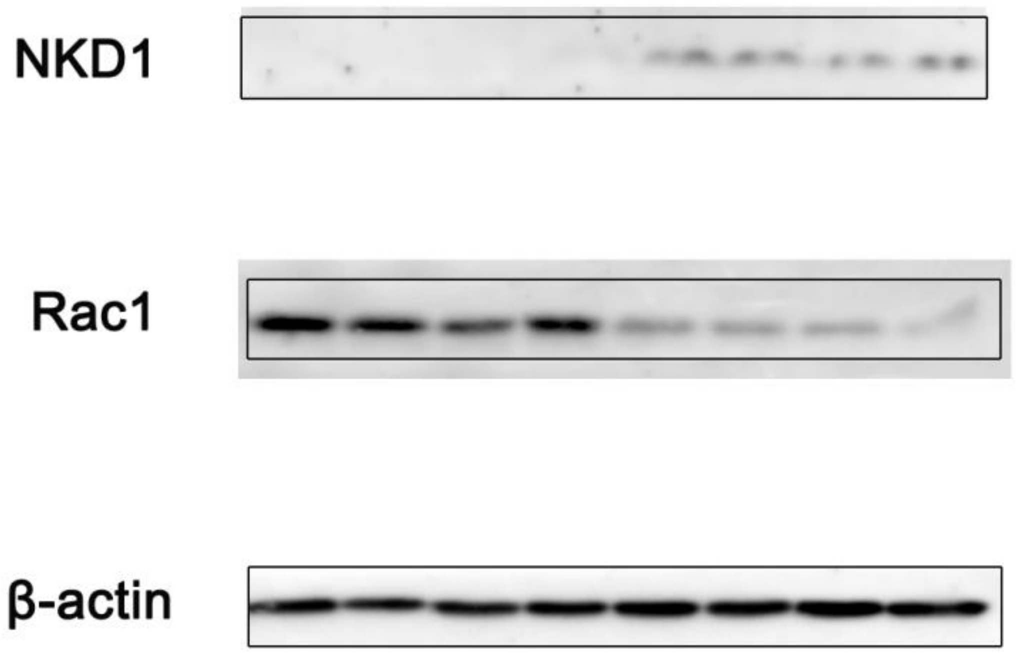

Supplement: Supplementary Information [file srep26971-s1.pdf]
